# Supplementary figures and images for: ENPP2 Promoter Methylation Correlates with Decreased Gene Expression in Breast Cancer: Implementation as a Liquid Biopsy Biomarker
Source: Int J Mol Sci. 2022 Mar 28;23(7):3717. doi: 10.3390/ijms23073717 (PMC8998992; doi:10.3390/ijms23073717)

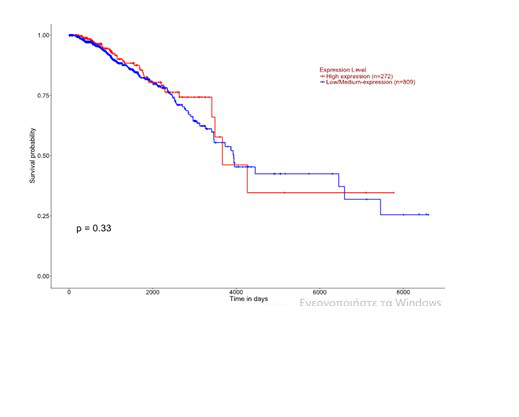

Supplement: Supplementary file 1 [file ijms-23-03717-s001.zip › Supplementary figure S1.jpg]

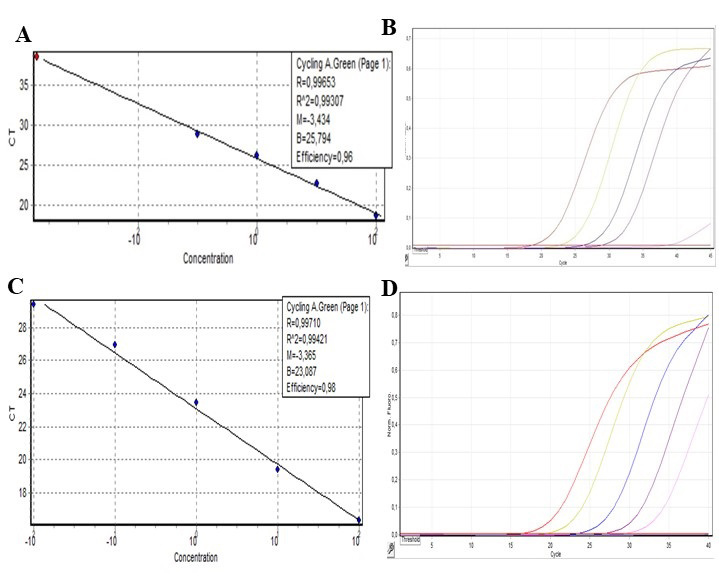

Supplement: Supplementary file 1 [file ijms-23-03717-s001.zip › supplementary figure S2.jpg]
